# Relationship Between Coronary Collateral Circulation and the Neutrophil-Percentage-to-Albumin Ratio in Patients with Chronic Coronary Syndrome

**DOI:** 10.3390/medicina61050779

**Published:** 2025-04-23

**Authors:** Zeki Cetinkaya, Yucel Yilmaz, Oguzhan Baran, Ozlem Secen, Mehmet Ali Gelen, Seyda Sahin, Ozkan Yavcin, Muhammed Ekmekyapar, Erkan Yıldırım, Saban Kelesoglu

**Affiliations:** 1 Department of Cardiology, Elazıg Fethi Sekin City Hospital, 23280 Elazıg, Türkiye; zeki.cetinkaya@sbu.edu.tr (Z.C.); ozllemsecen@hotmail.com (O.S.); maligelen4723@gmail.com (M.A.G.); seydshn.58@gmail.com (S.S.); ozkanycn@hotmail.com (O.Y.); 2 Department of Cardiology, Kayseri City Training and Research Hospital, University of Health Sciences, 38280 Kayseri, Türkiye; dryyilmaz@hotmail.com (Y.Y.); oguzhanbaran2009@hotmail.com (O.B.); 3 Department of Emergency Medicine, Elazıg Fethi Sekin City Hospital, 23280 Elazıg, Türkiye; m_ekmekyapar@hotmail.com; 4 Department of Cardiology, Fırat University Faculty of Medicine, 23119 Elazıg, Türkiye; drerkan23@yahoo.com; 5 Department of Cardiology, Erciyes University Faculty of Medicine, 38039 Kayseri, Türkiye

**Keywords:** coronary collateral circulation, neutrophil-percentage-to-albumin ratio, chronic coronary syndrome

## Abstract

*Background and Objectives:* The neutrophil-percentage-to-albumin ratio (NPAR) has been recognized as an independent risk factor for cardiovascular diseases. In our study, we investigated whether the NPAR is associated with the formation of coronary collateral circulation (CCC) in patients with chronic coronary syndrome (CCS). *Materials and Methods:* A total of 681 patients with CCS were included in this study. Of these patients, 571 had chronic total occlusion in at least one major vessel and developed collateral vessels. In total, 110 patients were in the control group, who had CCS but did not have complete occlusion in a major vessel and did not develop collateral vessels. Patients with collateral vessels on coronary angiography were divided into two groups according to the Rentrop score: poor CCC (Rentrop 0–1) and good CCC (Rentrop 2–3). Blood samples were taken for the NPAR and other biochemical parameters in all patients during hospitalization. The NPAR was calculated as the neutrophil-percentage-to-albumin ratio. *Results:* The group of patients with poor CCC had a higher white blood count (WBC), neutrophil, C-reactive protein (CRP), neutrophil–lymphocyte ratio (NLR), CRP/albumin ratio (CAR), and NPAR values than patients with good CCC (*p* < 0.001, for all). Multivariate logistic regression analysis showed that high NPAR levels were an independent predictor of poor CCC (OR: 2.79, 95% CI:1.7–4.6, *p* < 0.001), accompanied by neutrophil, CRP, CAR, and NLR levels. In the receiver operator characteristic curve (ROC analysis), the cut-off value for the NPAR to indicate poor CCC was 1.78 with a sensitivity of 76.6% and specificity of 81.4% (area under ROC curve = 0.804 95% CI (0.753–0.854), *p* < 0.001). *Conclusions:* We demonstrated that the NPAR may be an independent predictor of poor CCC development in clinical practice.

## 1. Introduction

Coronary collateral circulation (CCC) consists of a network of arteries and a collateral vascular system connecting them as of the embryological period [1]. In addition to age, genetic factors, and exercise, factors such as arteriogenesis and angiogenesis have been shown to affect the function of CCC [1,2]. CCC forms a natural bypass system of the heart’s blood vessels and is vital in individuals with coronary artery disease (CAD). This circulatory system can reduce ischemic damage by providing the heart with the blood it needs through alternative routes [1,3]. In addition to myocardial ischemia, inflammatory processes are also important in the development of CCC [4,5]. The induction of hypoxia activates endothelial cells, and the first inflammatory cells accumulate at arteriolar junctions, initiating the inflammatory cascade. Subsequently, the activation of a large number of transcription factors, chemokines, and growth factors takes place, and all these processes, which are part of inflammation, result in arteriogenesis and angiogenesis as well as the development/maturation of CCC [6].

Recently, there have been results suggesting that new inflammation markers derived from hematologic/biochemical parameters are effective in predicting the development of CCC, although there are not yet enough to be included in cardiology guidelines. The neutrophil-percentage-to-albumin ratio (NPAR) is a new marker used in the detection of cardiovascular diseases (CVDs). Furthermore, there is growing evidence showing the role of the NPAR in the pathophysiology of CVDs. Clarifying this relationship and understanding the predictive role of the NPAR on CCC in chronic coronary syndrome (CCS) patients is the primary focus of this study.

## 2. Materials and Methods

### 2.1. Study Population

In this retrospective study conducted between September 2021 and November 2023, 681 patients with CCS were included. A total of 110 of these patients were controls without coronary collateral circulation. The other patients were those with chronic total occlusion with major coronary artery occlusion and coronary collateral development with CCS. Patients with a history of acute coronary syndrome (ACS) within the last 3 months, chronic kidney disease (CKD) (glomerular filtration rate < 60 mL/min/1.73 m^2^), autoimmune disease, acute or chronic infectious diseases, history of coronary artery bypass grafting (CABG), severe valvular heart disease, congenital heart disease, decompensated heart failure (HF), malignancy, hematologic diseases, history of severe trauma or surgery within the last 3 months, immunosuppression, or hormone therapy were excluded.

### 2.2. Laboratory

Venous blood samples for complete blood count and biochemistry tests were collected from each participant in the morning when fasting. Blood samples were processed in the hospital laboratory as soon as possible after collection (within a maximum of 2 h). If, for any reason, they were not analyzed within this timeframe, the samples were refrigerated. All blood samples were analyzed within 24 h of collection. All routine biochemical tests were performed on an autoanalyzer (COBAS^®^ c701, Roche Diagnostics, Mannheim, Germany). Automated hematology analyzer equipment (Sysmex K-1000 Hematology Analyzer, Kobe, Japan) was used to test the hematological parameters. The neutrophil–lymphocyte ratio (NLR) was calculated as the number of neutrophils divided by the number of lymphocytes; the C-reactive protein (CRP)/albumin ratio (CAR) was calculated as CRP divided by albumin. By dividing the neutrophil percentage by the albumin level, the neutrophil percentage-to-albumin ratio (NPAR) was determined.

### 2.3. Coronary Angiography

Coronary angiography was performed using a catheter via the radial or femoral artery, depending on the operator’s preference. Blinded to the clinical and demographic information, two skilled interventional cardiologists evaluated each patient’s CAG image and CCC. If there were any discrepancies in the interpretation, a third reviewer who was blind to the readings of the first two reviewers decided on this matter.

The Rentrop classification, first described by Rentrop et al. [7], is the most commonly used angiographic grading system, although there are other approaches for evaluating CCC. Thus, the Rentrop classification was used in the current investigation to grade each patient’s collaterals. CCC filling was graded as follows: grade 0 indicated no collateral vessel filling, grade 1 indicated that the side branches of the artery were filled to form the epicardial segment, grade 2 indicated partial collateral vessel filling of the epicardial artery, and grade 3 indicated full collateral vessel filling of the epicardial artery. According to their CCC, patients were divided into two groups; poor CCC development was defined as Rentrop grades 0–1, while Rentrop grades 2–3 were defined as good CCC development.

Coronary lesions lasting more than three months that show either a stoppage of antegrade blood flow on CAG or minimal contrast penetration by the lesion without distal artery opacification are known as chronic coronary total occlusions (CTOs) [8]. All coronary lesions resulting in ≤50% stenosis in arteries larger than 1.5 mm were included in the SYNTAX score calculation. A group of two skilled interventional cardiologists used the most recent online version of the software on the website to generate the SYNTAX score [9] (https://syntaxscore.org/ (accessed on 1 April 2025)).

### 2.4. Statistical Analysis

Data analysis was performed using TurcosaAnalytics v1.0.0 software (Melikgazi, Kayseri, Turkey) and SPSS (Version 24 SPSS Inc., Chicago, IL, USA). Data were evaluated for normal distribution by the Shapiro–Wilk test and histogram Q-Q plots. When comparing poor and good CCC groups, the independent sample *t*-test was used for normally distributed parameters and the Mann–Whitney U-test was used for non-normally distributed parameters. Pearson’s χ^2^ test was used for categorical variables. ROC curve analysis was used to determine the sensitivity and specificity of the NLR, CAR, and NPAR values in the poor coronary collateral circulation group. CCC in patients with CCS was calculated with univariate analysis. For multivariate regression analysis, parameters with a *p* < 0.05 in univariate analysis were included in the model. Regression analysis was performed independently for each variable using inflammatory parameters to avoid multicollinearity.

## 3. Results

A total of 681 patients with CCS were included in this study. Patients with CCS were divided into three groups: a control group with CCS without collateral vessel development, patients with good CCC (Rentrop 2–3), and patients with poor CCC (Rentrop 0–1) according to the Rentrop classification. There were 110 patients in the control group, 434 patients in the good CCC group, and 137 patients in the poor CCC group. The mean ages of the patients were 54 (47–63), 53 (48–61), and 55 (49–64)

Demographic characteristics are summarized in Table 1. There was no significant difference between the groups in terms of hypertension (HT), hyperlipidemia, diabetes mellitus (DM) history, and body mass index (BMI), and the systolic/diastolic blood pressure arterial measurements were similar. There was no difference in terms of additional medications used between the control group, good CCC group, and poor CCC group (*p* < 0.05).

There was no statistically significant difference between the three groups in terms of biochemical parameters, including glucose, AST, ALT, creatine, albumin, and lipid values (*p* > 0.05). There was no significant difference between the two groups in terms of hematologic values, including hemoglobin, platelet, and lymphocyte values (*p* > 0.05). The CRP value was higher in the group with poor collaterals (*p* < 0.001).

**Figure 1:**
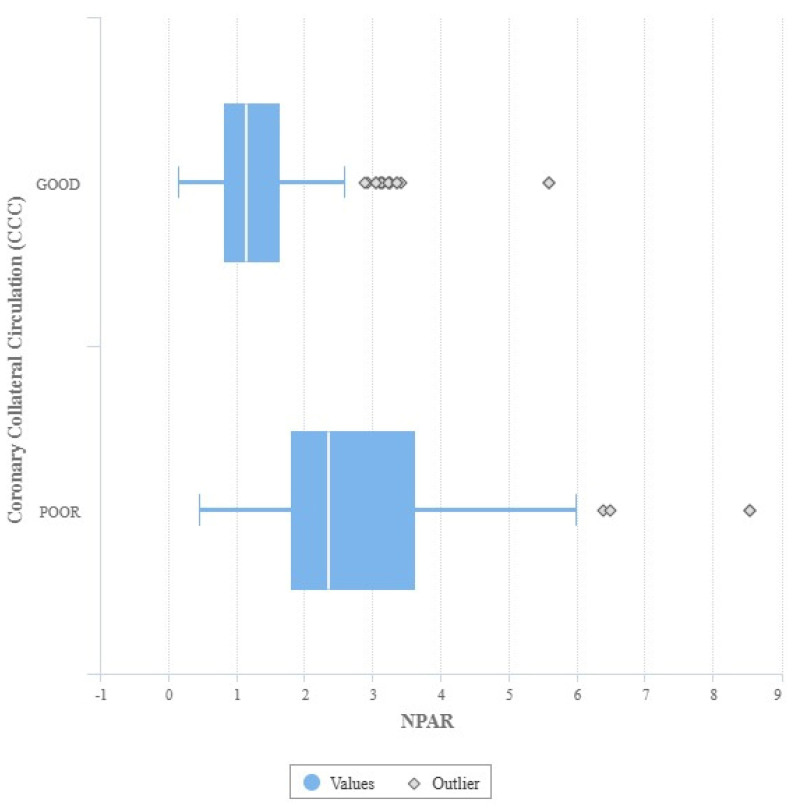
NPAR values of the coronary collateral circulation.

In patients with CCS, the group of patients with poor CCC had higher white blood cell count (WBC) levels (7.8 (5.8–9.21), 7.5 (5.76–9.18) vs. 12.3 (9.8–16.9), *p* < 0.001), and neutrophil count (4.42 (3.32–6.3), 4.32 (3.22–6.2) vs. 8.92 (6.38–13.72) *p* < 0.001), NLR (2.21 (1.46–3.3), 2.23 (1.48–3.21) vs. 4.6 (2.8–9.6), *p* < 0.001), CAR (0.9 (0.48–1.96), 0.93 (0.49–1.98) vs. 1.61 (0.95–2.18) *p* < 0.001), and NPAR (1.09 (0.79–1.59), 1.12 (0.81–1.61) vs. 2.34 (1.78–3.5) *p* < 0.001) (Figure 1) were higher (Table 2). When angiographic data were evaluated, the major coronary vessel in which the CTO was located and SYNTAX score were similar in both groups (Table 3).

In addition, the role of various risk factors in the occurrence of CCC was also evaluated by multivariate analysis. Multivariate logistic regression analysis was performed with values such as WBC, neutrophils, CRP, NLR, CAR, and NPAR, which were shown to be associated with poor CCC occurrence in univariate analysis. In multivariate logistic regression analysis, we found that high NPAR was an independent predictor of poor CCC outcome (OR: 2.79, 95% CI: 1.7–4.6, *p* < 0.001). CAR (OR: 1.56, 95% CI: 1.24–1.98, *p* < 0.001), NLR (OR: 1.11, 95% CI: 1.045–1.19, *p* < 0.001), CRP (OR: 1.12, 95% CI: 1.056–1.189, *p* < 0.001), and neutrophils (OR: 1.329, 95% CI: 1.162–1.52, *p* < 0.001) also predicted poor CCC (Table 4).

**Figure 2:**
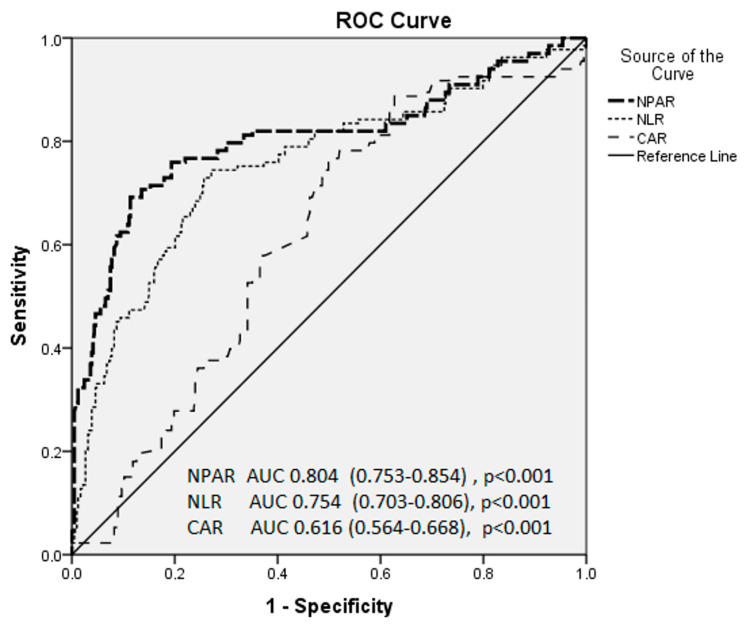
Receiver operating characteristic (ROC) curves for the inflammatory markers in predicting poor coronary collateral circulation in patients with chronic coronary syndrome.

In the ROC analysis, the cut-off value for the NPAR indicating poor CCC was 1.78 with a sensitivity of 76.6% and specificity of 81.4% (area under ROC curve = 0.804 95% CI (0.753–0.854), *p* < 0.001). The cut-off value for CAR indicating poor CCC was 0.948 with a sensitivity of 76.1% and specificity of 51% (area under ROC curve = 0.616 95% CI (0.564–0.688), *p* < 0.001). The cut-off value for NLR to indicate poor CCC was 3.08 with a sensitivity of 74.4% and specificity of 73.1% (area under ROC curve = 0.754 95% CI (0.703–0.806), *p* < 0.001) (Figure 2).

## 4. Discussion

This study evaluated the potential role of the NPAR in predicting poor CCC in patients with CCS. The NPAR has emerged as a potential predictor of poor CCC, and we observed that patients with weak CCC had high NPAR values. This relationship underscores the importance of systemic inflammation status in the formation and function of CCC.

When coronary artery disease is present, especially when there is severe stenosis, coronary collateral circulation plays a crucial compensatory role in maintaining myocardial perfusion. The variables affecting collateral expansion are still not entirely understood, however. Systemic inflammation’s impact on collateral formation has been extensively researched, and multiple studies have found a substantial correlation between inflammatory indicators and CCC [10,11]. According to this study, patients with poor CCC (Rentrop grades 0–1) had noticeably greater levels of inflammatory markers such as WBC, neutrophil count, NLR, CAR, and NPAR, which is consistent with the findings from other research [12,13,14].

Coronary collateral circulation plays a crucial role in limiting infarct size and preserving ventricular shape and function after ACS [15,16]. Many factors such as severity and duration of CAD, shear stress, endogenous mediators, DM, HT, hyperlipidemia, smoking, and type/duration of medications affect the development of CCC [17,18,19,20,21,22].

The neutrophil-percentage-to-albumin ratio is a recently developed marker and, like other similar inflammatory markers, was first used in studies to evaluate cancer patients. Xie et al. [23] reported that a high NPAR was an independent risk factor for postoperative complications and prognosis of patients with colorectal cancer. Feng et al. [24] claimed that NPAR is an independent variable for survival in patients with lung cancer. Recent studies have examined the prognostic significance of the NPAR in various CVDs. Elevated NPAR levels have been associated with increased mortality and adverse outcomes in patients with HF and CAD. Karaca et al. [25] claimed that the NPAR is a newly identified promising inflammatory biomarker for predicting one-year major adverse cardiac and cerebrovascular events (MACCEs) in non-ST elevation myocardial infarction (NSTEMI) patients undergoing revascularization therapy. This suggests that NPAR may be a useful prognostic indicator in this patient population. Cui et al. [26] found an independent association between the NPAR and in-hospital mortality in ST elevation myocardial infarction (STEMI) patients. Zhao et al. [27] found that the NPAR was independently associated with the severity of coronary atherosclerosis in patients with chronic kidney disease. Zang et al. [28] found that elevated NPAR levels were an independent predictor of coronary slow flow phenomenon in patients with ischemia with no obstructive coronary arteries (INOCA). Wang et al. [29], in a retrospective study of 622 HF patients, found that high NPAR levels during hospitalization were independently associated with 90-day, 1-year, and 2-year all-cause mortality. Furthermore, this study concluded that the NPAR can be used as a valuable marker for risk stratification in HF patients. Furthermore, a study conducted between 2005 and 2016 analyzed data from the U.S. National Health and Nutrition Examination Survey (NHANES) and found that elevated NPAR levels were independently associated with all-cause mortality in community-dwelling individuals with HF. This highlights the potential of the NPAR as a prognostic marker outside of hospitalized patients [30]. As a result of all these studies, the NPAR has been considered a promising prognostic marker for predicting mortality, especially in patients with HF and CAD. Studies have also been conducted to determine the prognostic or diagnostic value of the NPAR in CVDs other than CAD and HF. Cai et al. [31], in their study, reported that NPAR values were positively associated with cardiovascular mortality in patients with atrial fibrillation. In our study, we found that NPAR was higher in patients with poor CCC development compared to those with good CCC development. There is no consensus on which inflammatory marker is better to use in the evaluation of CCC development. Previous studies published in the literature support that inflammation suppresses CCC development and that systemic inflammatory markers may influence this process.

Inflammatory indices in the evaluation of CCC were first used in 2013 by Ayhan et al. [32] in a study in which NLR and mean platelet volume (MPV) were evaluated. In this study, they reported that both were associated with poor CCC. In their studies, Açar et al. [33] focused on the platelet-to-lymphocyte ratio (PLR), Kelesoglu et al. [14,34] CAR and the systemic immune inflammation index (SII), Yilmaz et al. [13] the pan immune inflammation value (PIV), and Toprak et al. [35] the uric acid/albumin ratio. In all the studies mentioned above, increased index ratios were correlated with poor CCC. In our study, NLR and CAR values were found to be high in the patient group with poor collateral circulation in patients with chronic total occlusion, and a statistically significant difference was found from the other groups.

The mechanisms involved in the adequate development of CCC have not been fully elucidated. However, there is increasing evidence that the inflammatory cascade is one of the important hairpins. The prognostic value of the NPAR can be attributed to the combined effects of neutrophils and albumin. Neutrophils play an important role in innate inflammation and have been associated with various CVDs such as CAD and myocardial infarction [36]. Neutrophils are also believed to play an important role in the early/initiation phase of arteriogenesis. It is believed to enhance arteriogenesis by directly affecting endothelial cells by binding to certain kinase proteins and resulting in nitric oxide species (NOS)-mediated vasodilation and by inducing activated neutrophils to express and release growth factors [37,38]. Neutrophils are also an important source of circulating reactive oxygen species (ROS) [39]. Albumin is considered a negative acute-phase reactant, and a decrease in albumin levels will result in an increase in blood viscosity and therefore the increased oxidation of low-density lipoprotein cholesterol (LDL-C) and hence increased foam cell formation and inflammation [40]. Vascular endothelial damage is exacerbated by the mediators and growth factors released. However, low levels of albumin may reduce the production of angiogenesis-promoting factors such as vascular endothelial growth factor (VEGF), leading to inadequate collateral circulation. Albumin also has anti-inflammatory, antioxidant, anticoagulant, and antiplatelet aggregation activity [41]. The potential role of low serum albumin levels in insufficiency in the development of CCC may point to its reduced antioxidant, anti-inflammatory, and platelet aggregation effects. These are important factors in the development of CCC. In our study, in the patient group with chronic total occlusion who developed poor collateral circulation, the neutrophil count was found to be high and the albumin count was found to be low, and a statistically significant difference was observed from the other two groups.

The association between CVDs and the NPAR is an area of increasing research, although it is not yet as firmly established as other biomarkers, such as the NLR. Preliminary findings suggest that the NPAR might serve as a potential inflammatory marker for CVDs. The present study suggests that elevated NPAR levels may be a more sensitive parameter in determining the risk of poor CCC.

## 5. Conclusions

The results of our study could be potentially useful in clinical applications. It is possible that a simple, inexpensive, and easily calculable parameter such as the NPAR could be used for the early detection of patients at risk of poor CCC. This could allow for the early identification of patients at high risk and the implementation of appropriate treatment strategies.

Although there has been interest in the NPAR as a biomarker, proposed as an indicator of systemic inflammation, a critical factor in the development and progression of CVDs such as ACS and HF, studies examining its direct association with CVDs are still limited. Larger-scale, multicenter, prospective studies are needed to confirm its diagnostic and prognostic value and to establish standardized NPAR thresholds for clinical practice.

### 5.1. Limitations

First, this study was single-center and conducted on a relatively small group of patients. Furthermore, a retrospective method was used instead of a prospective design. Therefore, larger-scale, multicenter, and prospective studies are needed to confirm our results.

### 5.2. Simple Summary

We investigated whether the poor or good bridging vessel developed by the heart in people with ischemic heart disease in cases of coronary artery occlusion could be related to a new inflammatory marker called NPAR. We observed that NPAR values were high, especially in people with weak bridging vessels.

## Figures and Tables

**Table 1:** Demographic characteristics of the study population.

Coronary Collateral Circulation				
Variables	Control Group (n = 110)	Poor (n = 137)	Good (n = 434)	*p* Value
Age (years)	54 (47–63)	55 (49–64)	53 (48–61)	0.081
Male gender (n, %)	79 (72%)	98 (71.5%)	329 (76%)	0.315
Diabetes mellitus (n, %)	40 (37%)	57 (41.9%)	148 (34.3%)	0.21
Hypertension (n, %)	53 (48.1%)	71 (52.2%)	212 (49.1%)	0.56
Dyslipidemia (n, %)	16 (14.6%)	25 (18.2%)	60 (13.8)	0.205
Smoking (n, %)	38 (34.6%)	56 (40.9%)	139 (32.1%)	0.063
BMI (kg/m^2^)	27.1 ± 3.9	28.4 ± 4.5	27.9 ± 4.6	0.346
Systolic blood pressure (mmHg)	122.6 ± 13.4	123.4 ± 18.9	121.8 ± 12.8	0.152
Diastolic blood pressure (mmHg)	72.9 ± 8.4	73.5 ± 8.9	74.1 ± 8.5	0.31
Heart rate (beats/min)	74.6 ± 12.9	76.7 ± 13.1	75.9 ± 13	0.642
Previous medications, n (%)				
ASA, n (%)	43 (38.9%)	54 (39.4%)	175 (40.5%)	0.76
Β-blocker	15.6 (14.2%)	27 (19.7%)	107 (24.8%)	0.124
Statin	9 (8.6%)	16 (11.7%)	46 (10.7%)	0.63
ACEI/ARB, n (%)	33 (30.1%)	53 (38.7%)	186 (43.1%)	0.167
Aldosterone antagonists	1 (1.2%)	4 (2.9%)	19 (4.4%)	0.129
Calcium channel blockers	10 (9.6%)	14 (10.2%)	55 (12.7%)	0.212
nitrate	1 (1.1%)	4 (2.9%)	9 (2.1%)	0.117
Clopidogrel	3 (3.1%)	15 (10.9%)	45 (10.5%)	0.08

Abbreviations: BMI, body mass index; ASA, acetylsalicylic acid; ACEI, angiotensin converting enzyme inhibitor; ARB, angiotensin receptor blocker.

**Table 2:** Laboratory findings of the study population.

Coronary Collateral Circulation				
Variables	Control Group (n = 110)	Poor (n = 137)	Good (n = 434)	*p* Value
Glucose (mg/dL)	147.8 ± 64.6	161.29 ± 88.6	149.5 ± 68.5	0.109
Creatinine (mg/dL)	0.94 ± 0.21	0.923 ± 0.22	0.95 ± 0.2	0.13
AST (U/L)	18 (15–21)	19 (16–22)	18 (14–22)	0.184
ALT	18 (16–23)	20 (15–27)	19 (14–25)	0.186
Total cholesterol (mg/dL)	171 (139–208)	166.5 (142–196)	175 (141–210)	0.281
High-density lipoprotein cholesterol (mg/dL)	37 (32–45)	38 (30–44)	36 (31–43)	0.714
Low-density lipoprotein cholesterol (mg/dL)	112 (82–138)	109 (83–125)	114 (85–146)	0.36
Triglyceride (mg/dL)	116 (80–166)	111 (75–178)	114 (78–163)	0.54
Hemoglobin (mg/dL)	13.9 (12.7–15.2)	14.2 (12.8–15.1)	14.3 (13.3–15.3)	0.121
Platelets (10^3^/µL)	221 (185–259)	224 (186–258)	219.5 (187.25–261)	0.841
WBC (10^3^/µL)	7.8 (5.8–9.21)	12.3 (9.8–16.9)	7.5 (5.76–9.18)	<0.001
Neutrophil (10^3^/µL)	4.42 (3.32–6.3)	8.92 (6.38–13.72)	4.32 (3.22–6.2)	<0.001
Lymphocyte (10^3^/µL)	1.82 (1.36–2.49)	1.78 (1.34–2.4)	1.85 (1.39–2.52)	0.39
CRP (mg/L)	3.86 (1.7–7.9)	5.66 (4–8)	3.89 (1.8–8)	<0.001
Albumin (g/L)	3.7(3.4–4.1)	3.4 (3.3–4.1)	3.8 (3.5–4.1)	0.07
NLR	2.21 (1.46–3.3)	4.6 (2.8–9.6)	2.23 (1.48–3.21)	<0.001
CAR	0.9 (0.48–1.96)	1.61 (0.95–2.18)	0.93 (0.49–1.98)	<0.001
NPAR	1.09 (0.79–1.59)	2.34 (1.78–3.5)	1.12 (0.81–1.61)	<0.001

Abbreviations: AST, aspartate aminotransferase; ALT, alanine aminotransferase; WBC, white blood cell; CRP, C-reactive protein; NLR, neutrophil/Lymphocyte ratio; CAR, CRP/albumin ratio; NPAR, neutrophil-percentage-to-albumin ratio.

**Table 3:** Angiographic data.

	Coronary Collateral Circulation		
	Poor (n = 137)	Good (n = 434)	*p* Value
Rentrop collateral grades:			
0	76 (55.4%)	0 (0%)	<0.001
1	61 (44.6%)	0 (0%)	
2	0 (0%)	248 (51.14%)	
3	0 (0%)	186 (42.8%)	
Position of chronic total occlusion:			
Left anterior descending coronary artery	49 (35.8%)	144 (33.2%)	0.841
Left circumflex coronary artery	43 (31.4%)	145 (33.4%)	
Right coronary artery	45 (32.8%)	145 (33.4%)	
SYNTAX score	21.79 ± 7.85	22.94 ± 8.1	0.151

**Table 4:** Univariate and multivariate predictors of poor coronary collateral circulation in patients with chronic coronary syndrome.

	Univariate Analysis			Multivariate Analysis		
	Odds Ratio	95% CI	*p* Value	Odds Ratio	95% CI	*p* Value
WBC	1.368	1.285–1.457	<0.001	1.103	0.991–1.227	0.074
Neutrophil *	1.422	1.325–1.527	<0.001	1.329	1.162–1.520	<0.001
CRP **	1.053	1.006–1.103	0.028	1.12	1.056–1.189	<0.001
NLR *	1.278	1.205–1.356	<0.001	1.11	1.045–1.19	<0.001
CAR **	1.360	1.123–1.647	0.002	1.56	1.24–1.98	<0.001
NPAR *	3.73	2.87–4.85	<0.001	2.79	1.7–4.6	<0.001

Abbreviations: WBC, white blood cell; CRP, C-reactive protein; NLR, neutrophil/Lymphocyte ratio; CAR, CRP/albumin ratio; NPAR, neutrophil-percentage-to-albumin ratio. *, ** These parameters were not entered into the model in order to prevent multicollinearity.

## Data Availability

The data can be obtained from the corresponding author upon request. The code for this article is available from the first author page at: https://dosya.sbu.edu.tr/CV/ZEKICETINKAYA_2562.pdf (accessed on 15 February 2025).
